# What Becomes of the Decidua in Abortion?

**Published:** 1866-03

**Authors:** Eben Hillyer

**Affiliations:** Prof. of Physiology in the Atlanta Medical College


### ARTICLE II.

*What becomes of the Decidua in Abortion?* By EBEN HILLYER, M. D.,
Prof. of Physiology in the Atlanta Medical College.

There has always been much uncertainty and mystery as to the formation of the deciduous membrane in fœtal development; some gentlemen have even gone so far as to doubt its existence at all. They should admit the fact of its existence, even though they deny the theory of its formation, as it is one of the membranes enveloping the fœtus when it is delivered from the mother by the process of parturition. It is found, naturally, to consist of two layers, both together forming a tough vascular membrane—they are closely adherent, and are quite difficult of separation. The chorion and amnion are not supplied with red vascular vessels, while the decidua is well supplied with red blood. The inner membrane is called the *decidua reflexa*, the outer membrane the *decidua vera*.

It was formerly supposed by Hunter and others, that this membrane was formed by an exudation of plastic lymph, thrown out upon the inner surface of the womb, which becomes organized similar to the pseudo membranous exudation of croup—that the openings of the Fallopian tubes were closed by the formation, and that as the ovum came down out of the tube into the womb, and pushed forward the membranous expansion before it, that portion immediately covering the ovum becoming the decidua reflexa, and that lining the inside of the womb being the dicidua vera. For a long time this was supposed to be the correct solution of the development of the decidua. This membrane is undoubtedly formed independently of the ovum, as is proven by the fact that it exists in cases of extra uterine pregnancy.

The theory of the formation of the decidua, as now understood and taught by most of the physiologists of the present day is altogether different from the theory of Mr. Hunter just stated. The modern theory, as established by the researches of Sharpey, Weber and others, is as follows: That at the time an ovum is fecundated, the mucus membrane of the womb, which is tubular in its structure, somewhat like that of the stomach, becomes thickened, hypertrophied, and thrown into rugous folds. This increased development is quite extensive, the spaces between the ridges forming deep furrows or sulchi, on account of which the orifices of the Fallopian tubes are not closed up. When the fecundated ovum passes down out of the tube into the womb, it becomes lodged in one of these sulchi or furrows. The peculiar structure of this lining membrane of the uterus is not particularly altered, but simply increased in development; at the same time, there exuded into the uterine cavity a peculiar fluid in which the tuft or villi of the chorion hang out and imbibe from it nourishment for the embryo within, until further provision is made by attachment. These villi of the chorion, also, where they are in contact with the mucous membrane next to womb and the side of the ridges, insinuate themselves into the tubules of the membrane: this is for further nourishment. The lodgment is generally in the fundus of the womb, or very near the mouth of the Fallopian tube. The sides of the ridge around the ovum continues to grow up and enclose it, so that shortly it is entirely covered in; it is only the two ridges, between which the ovum lies, now continue to increase in development; the other portions of the mucous surface from this time on gradually go back toward the normal condition; but not entirely so. The villi of the chorion are now inserted all around into the tubular structure of the mucous surface. As the ovum increases in growth, the villi, which are on the side next to and in contact with the womb, increase in development, and have blood vessels going with them; those on the outer side, or the side which looks into the cavity of the womb, have no blood vessels. After a short period, the villi on this side begin to be absorbed, and finally disappear; and this portion of the covering of the ovum, which is toward the cavity of the uterus, becomes the *decidua reflexa;* that portion of the thickened mucous membrane lining the rest of the uterine superficies, becomes the *decidua vera.* At this early period the decidua reflexa has the mark or cicatrix where the two ridges have joined over the ovum. After a time the villi which are attached on the womb side are developed into the placental structure, and this portion of the mucous membrane becomes the true placental substance. At a later period of pregnancy the decidua reflexa has been pushed further and further into the womb, until it comes in contact with the decidua vera, and then it presents the appearance which we see at full term. It will be observed, finally, that this membrane is the mucous membrane of the womb, which is thus altered and turned into one of the membranes covering the folds, and is thrown off at each pregnancy, *hence its name.*

It is the object of this article, after thus giving the description of the deciduous formation, to enquire into its use, and the purpose it subserves in the development of the embryo; *also, what becomes of it* in cases of abortion in the early months of pregnancy. Its function is undoubtedly to furnish a means of immediate support and nutrition for the ovum when it enters the womb, or as soon as it has been fecundated. We have already seen how the tufts of the chorion are inserted into the mucous tubules, and it is from this source that the support is derived, until the placenta is formed. It is a considerable period until this formation, and attachment (placental) is established; but by the peculiar condition which the lining surface of the womb is thrown into, there is a soft and well prepared structure, favorable to giving up fluids by absorption, for the ovum to lodge in. After the placental arrangement is completed, it is no longer of any use, and is then developed into the double membrane which we find at maturity of the embryo.

Should abortion occur at the end of the first month, it is an interesting enquiry what becomes of the decidua; is the mucous surface of the womb now exfoliated and thrown off? This would be rather a hard thing to conceive possible, and we are led to think that it is not. At this time the ovum would escape from the sulchi and be expelled, only enveloped in the chorion and amnion, and floating in the amnionic fluid. I have seen cases of abortion at this period, and the decidua did not appear. At such time, the mucous membrane returns to its normal condition, and does not become deciduous. Abortion occurring at a later period, say at the second month, we might have the whole mass coming away, and we might not. I have had it both ways. At this period, when the ovum comes only covered by the chorion and amnion, it is very perplexing to tell what becomes of the deciduous mass. Were it to all come together, we would find the ovum lodged in the top, or upon one side of the deciduous body. I have had the ovum to come first, and sometime afterwards the decidua. Sometimes it was not known when the ovum came away, and the decidua alone was all the remains of the fœtal existence which could be found. In this case, it presents very much the appearance of a mole, or a blasted ovum, and I mistook the first I ever saw for a formation of this kind. I think even at the end of the sixth week, or at the end of two months, when the decidua comes away, it is the exception, and does not often happen. I am led to this belief for the reason that I have met with a number of cases in which the ovum alone was all that I could get away, although I several times made continued but prudent efforts to do so. In these cases I expected the deciduous mass to come away by disintegration, with the secretions which follow the delivery, with much feter and offensive discharge, as is always seen when any of the secundines are left; but, to my surprise, no such disagreeable or offensive discharge occurred, nor was it unnaturally continued. In two cases I could plainly feel the deciduous mass inside of the cervix, and it felt as if there could be no difficulty in detaching it and bringing it away; but it provoked such hemmorrhage that it was plainly prudent to desist, and let it come away in its own time. But, as I said, in several cases it did not come away either whole or in part by the process of disintegration. What becomes of it? It seems unreasonable to suppose that it could be absorbed; but nevertheless, I believe that, as this is the true mucous lining surface of the womb in a state of hypertroply, that its excess of structure is absorbed, and it, at this early period, returns to its normal condition, as it undoubtedly does in abortion at the first month.

On the other hand, whenever it has come away in the cases referred to, there has been a great deal of hemmorrhage, which was the result of the unnatural tearing away of the mass from its attachment to the surface of the womb. In those cases where the detachment and exfoliation at these early periods is evidenced by alarming hemmorrhage, it is decidedly proper to use the tampon; for hemmorrhage will continue until separation and expulsion is complete. No danger can result from the tampon now, as the womb cannot hold, with the ovum blood, enough to cause danger by its loss. When the tampon is removed, the cervix being dilated and relaxed, the ovum, decidua and all come together. Now, where the ovum is lodged in the fundus, or in the top of the deciduous mass, the whole generally does, and necessarily must, come together; for the decidua reflexa is now so firmly joined in front of it, that it cannot get through. It seldom does, we would think, come down between the decidua vera and the side of the womb. We have already found that at full term the decidua's formation is thinned out to a membrane, and lost its character as a part of the structure of the womb, as it is long before this time exfoliated, and a new internal surface formed to the uterus in its stead. It is then a membranous expansion from the circumference of the placenta, and is expelled along with the other secundines. After the third or fourth week, it must always be expelled, and just at the period when it is too far gone into the membranous state to return to its normal condition. There will be (should abortion occur at this time) dangerous and alarming hemmorrhage from the free exposed surface of the womb, from which the separation has just occurred.

